# Draft Genome Sequences of Five Clinical Strains of *Brucella melitensis* Isolated from Patients Residing in Kuwait

**DOI:** 10.1128/genomeA.01144-16

**Published:** 2016-11-03

**Authors:** Mohd Wasif Khan, Nazima Habibi, Faraz Shaheed, Abu Salim Mustafa

**Affiliations:** a OMICS Research Unit, Research Core Facility, Health Sciences Centre, Kuwait University, Kuwait; b Department of Microbiology, Faculty of Medicine, Kuwait University, Kuwait

## Abstract

Human brucellosis is a neglected and underrecognized infection of widespread geographic distribution. Brucellosis is present on all inhabited continents and endemic in many areas of the world, including Kuwait and the Middle East. Here, we present draft genome assemblies of five *Brucella melitensis* strains isolated from brucellosis patients in Kuwait.

## GENOME ANNOUNCEMENT

Brucellosis is a highly infectious zoonotic disease caused by the organisms of genus *Brucella* (1). Among the 10 known species of *Brucella* (2), *Brucella melitensis* is the most pathogenic organism and a biological threat to humans (3). Genotyping of *B. melitensis* isolates is important for contact tracing and epidemiological surveillance in regions endemic for the organism (4). Whole-genome sequencing, by using next-generation sequencing (NGS) technologies, is emerging as a rapid method for the genetic characterization of *B. melitensis* (5, 6). NGS-based assays are capable of identifying genetic variations in the form of single nucleotide polymorphisms (SNPs) and indels (5, 6). In order to identify genetic variations in strains from Kuwait, we did whole-genome sequencing of five *B. melitensis* strains isolated from patients residing in Kuwait. The sequence data were analyzed to determine the number of SNPs in the genomes.

Five clinical isolates of *B. melitensis* were grown on culture plates, and single colonies were suspended in saline. The bacterial suspensions were heated at 95°C for 10 min, and DNA was purified using the QIAamp DNA minikit (Qiagen, Hilden, Germany). DNA libraries were prepared using the Nextera XT DNA library preparation kit (Illumina, San Diego, CA). The genomic DNA was sequenced using Illumina MiSeq paired-end (2 × 150 bp) sequencing technology. The data quality was checked with FastQC (7). Reads were trimmed and quality filtered using FASTX-Toolkit (http://hannonlab.cshl.edu/fastx_toolkit/). The *de novo* assembly was performed using Velvet 1.2.10 (8). The best Velvet assembly was merged with SPAdes assembly by using SPAdes 3.8 (9). Quast was used to check the assembly quality (10). After ordering the obtained contigs against *B. melitensis* bv. 1 strain 16M using the Mauve contig aligner (11), each draft assembly was submitted to the NCBI for annotation with PGAP 3.3. SNPs were detected relative to the genome of reference strain *B. melitensis* bv. 1 strain 16M (GenBank assembly accession no. GCA_000007125.1) using BioNumerics 7.6 (Applied Maths, Belgium). All the assemblies have 57.2% G+C content and 3 rRNAs. Other assembly/genome characteristics, i.e., mean coverage, *N*_50_ contig length, number of contigs, assembly size, and number of genes, tRNAs, and SNPs are provided in Table 1. In-depth comparative analyses of these genomes are under way and will be published in an upcoming manuscript.

**TABLE 1:** Summary characteristics of whole-genome assemblies of five clinical *B. melitensis* strains isolated in Kuwait

Strain	Mean coverage (×)	*N*_50_ contig length (bp)	No. of contigs	Assembly size (bp)	No. of genes	No. of tRNAs	No. of SNPs	Accession no.
KU/RCF-03	43.0	189,106	43	3,283,635	3,143	52	1,042	LDTY00000000
KU/RCF-31	40.0	176,881	49	3,289,202	3,151	51	1,493	LDTX00000000
KU/RCF-64	52.6	268,511	40	3,290,511	3,154	52	1,501	LDTW00000000
KU/RCF-84	69.0	293,285	34	3,289,577	3,150	52	1,505	LDTV00000000
KU/RCF-96	67.2	195,560	41	3,289,620	3,150	52	1,487	LAQM00000000

### Accession number(s).

All the genome sequences were submitted to NCBI under BioProject PRJNA278809 and are available with accession numbers listed in Table 1.
